# MiR-214 prevents the progression of diffuse large B-cell lymphoma by targeting PD-L1

**DOI:** 10.1186/s11658-019-0190-9

**Published:** 2019-12-04

**Authors:** Jing-Ran Sun, Xiao Zhang, Ya Zhang

**Affiliations:** 1 Liaocheng Central Blood Station, 75 Huashan Road, Liaocheng, Shandong 25200 People’s Republic of China; 2 Department of Clinical Laboratory, Liaocheng People’s Hospital, 67 Dongchang West Road, Liaocheng, Shandong 25200 People’s Republic of China; 3 Department of Gynecology and Obstetrics, Liaocheng People’s Hospital, 67 Dongchang West Road, Liaocheng, Shandong 25200 People’s Republic of China

**Keywords:** Diffuse large B-cell lymphoma, miR-214, PD-L1, Proliferation, Invasion, T cells

## Abstract

**Objective:**

We explored the role and mechanism of miR-214 involvement in the progression of diffuse large B-cell lymphoma (DLBCL).

**Methods:**

The expression levels of miR-214 and PD-L1 in human DLBCL cell lines and in tissue samples from patients with DLBCL were determined using quantitative RT-PCR. The dual-luciferase reporter assay was employed to determine the correlation between the expressions of miR-214 and PD-L1. Cell viability, invasiveness and apoptosis were respectively examined in cells of the DLBCL line OCI-Ly3 using CCK-8, transwell and flow cytometry assays. The expression level of PD-L1 was determined via immunoblotting. Inflammatory cytokine secretion was determined via enzyme-linked immune sorbent assay (ELISA).

**Results:**

miR-214 was downregulated and PD-L1 was upregulated in DLBCL tissues and cell lines in comparison to normal adjacent tissues or normal B-cell. This indicates a negative correlation in the expression levels. Overexpression of miR-214 inhibited cell viability and invasion and induced apoptosis of OCI-Ly3 cells. Moreover, miR-214 was shown to target PD-L1 mRNA by binding to its 3′-untranslated region (UTR). Knockdown of PD-L1 attenuated the malignant phenotype of OCI-Ly3 cells. Overexpression of miR-214 inhibited tumor growth by targeting PD-L1 in vivo.

**Conclusion:**

By targeting PD-L1, miR-214 regulates the progression of DLBCL in vitro and in vivo.

## Background

Diffuse large B-cell lymphoma (DLBCL) is a highly heterogeneous malignant lymphoma [1, 2]. Despite the current greater understanding of DLBCL pathological subtypes and the effectiveness of rituximab-based chemoimmunotherapy, 35–40% of patients show diminished treatment efficacy due to the rapid emergence of drug resistance [3]. To improve survival rates, there is an urgent need for an in-depth understanding of DLBCL pathogenesis and the mechanisms that lead to drug resistance.

Previous studies found that microRNAs (miRNAs) participate in the regulation of cancer cells’ malignant biological behavior [4, 5]. For example, miR-214 has been confirmed to act as a tumor suppressor gene in the development of various malignant tumor types, including colon [6], breast [7], ovarian [8], non-small cell lung [9] and gastric cancer [10], by decreasing cell proliferation and invasion and increasing apoptosis rates. Furthermore, miR-214 is downregulated in DLBCL tissues [11]. In addition, elevated miR-214 was associated with a superior outcome for this cancer type [12]. This led us to speculate that overexpression of miR-214 may inhibit the malignant behavior of DLBCL cells by targeting a downstream gene, but the mechanism details are not clear. The role of miR-214 in DLBCL progression is the focus of this study.

Programmed death ligand-1 (PD-L1) is a commonly found negative immunoregulatory protein [13]. It has great significance in the avoidance harmful autoimmune reactions [3]. Many studies have found that PD-L1 expression significantly increases in various malignant tumor tissues and cell lines, inducing the increase of immune cell apoptosis, which is an important regulatory mechanism for tumor immunosuppression [14, 15]. PD-L1 is widely distributed in various organs, circulatory systems and tumor tissues, with its distribution mainly modulated by the molecular microenvironment (IFN-γ, miRNAs, etc.) in which the cells are located [16, 17].

Several studies have confirmed that PD-L1 targeting underlies the involvement of multiple miRNAs in regulating the development of malignant tumor types, including gastric cancer [18], melanoma [19] and DLBCL [20]. He et al. reported that overexpressed miR-195 targets PD-L1 to attenuate DLBCL progression by decreasing the immune escape of DLBCL cells [21]. However, further information on the role of PD-L1 in the malignant behavior of DLBCL cells is needed.

This study investigated how the miR-214–PD-L1 axis is involved in the regulation of tumor growth and the function of T cells in vitro and in vivo. In addition, a new therapeutic biomarker for DLBCL treatment in the clinic is assessed.

### Materials and methods

#### Tissue specimen

DLBCL tissues and paired normal adjacent tissues were collected from patients (*n* = 15) at Liaocheng People’s Hospital. The clinical specimens were immediately frozen at − 80 °C. None of the patients had received chemotherapy or radiotherapy prior to surgery. All the patients signed informed consent. Approval for the study was obtained from the Ethical Committee of Liaocheng People’s Hospital and all procedures complied with the guidelines and principles of the Declaration of Helsinki.

### Cell culture

Human DLBCL cell lines (OCI-Ly3, SU-DHL-2 and OCI-Ly10), a normal B-cell line (NBC) and HEK-293 T cells were purchased from the Shanghai Institute for Biological Sciences of the Chinese Academy of Sciences. Cells were cultured according to the manufacturer’s instructions in Dulbecco’s modified Eagle’s medium (DMEM; Gibco) supplemented with 1% penicillin–streptomycin and 10% fetal bovine serum (FBS; Thermo Fisher Scientific) at 37 °C in a 5% CO_2_ atmosphere.

### T cell and OCI-Ly3 cell co-culture

T cells were obtained from the peripheral blood of healthy donors and DLBCL patients. A total of 2 × 10^5^ T cells/ml were seeded in 96-well plates cultured in 5% CO_2_ at 37 °C. T-cell Activation (Thermo Fisher Scientific) was added according to the manufacturer’s protocol. OCI-Ly3 cells were co-cultured with the activated T cells at a ratio of 9:1 for 24 h before transfection with miR-214 inhibitor and PD-L1 siRNA or with PD-L1 siRNA alone.

### Cell transfection

OCI-Ly3 cells were seeded in 6-well plates with 2 × 10^5^ cells per well and then incubated for 24 h. The miR-214 mimic and inhibitor, PD-L1 siRNA (si-PD-L1), and control were transfected into OCI-Ly3 cells with Lipofectamine 3000 reagent and Opti-MEM medium (Invitrogen) according to the manufacturer’s protocols. The PD-L1 siRNA, miR-214 mimic and inhibitor, and control (blank plasmid) were purchased from Tolo Biotech. The sequences were miR-214 mimic, 5′-ACAGCAGGCACAGACAGGCAGU-3′; miR-214 inhibitor, 5′-ACUGCCUGUCUGUGCCUGCUGU-3′; and control, 5′-UUGUACUACACAAAAGUACUG-3′.

### Real time quantitative polymerase chain reaction (RT-qPCR)

Total RNA was extracted from clinical specimens and cell lines using Trizol reagent (QIAGEN) according to the manufacturer’s instructions. For mRNA detection, RNA samples were reverse-transcribed into cDNA using a PrimeScript RT reagent kit with gDNA Eraser (TaKaRa). Quantitative real-time PCR was performed with GoTaq qPCR Master Mix (TaKaRa) using the CFX96 Sequence Detection System (Bio-Rad). For miRNA detection, RNA samples were reverse transcribed using a Mir-X miRNA First-Strand Synthesis kit (TaKaRa) and real-time PCR was done with an Applied Biosystems 7300 Real-Time PCR system. The primer sequences are given in Table 1. U6 and GAPDH were respectively used as the endogenous reference for miRNA and mRNA. Real-time PCR was performed in triplicate.

**Table 1:** Name and sequences of the primers

Name	Primer sequences
miR-214	F: 5′ – CAATACTGACAGCAGGCACA – 3′ R: 5′ – TATGGTTGTTCACGACTCCTTAC – 3′
U6	F: 5′ – CTCGCTTCGGCAGCACA – 3′ R: 5′ – AACGCTTCACGAATTTGCGT – 3′
PD-L1	F: 5′ – GGTGAGGATGGTTCTACACAG – 3′ R: 5′ – GAGAACTGCATGAGGTTGC – 3′
GAPDH	F: 5′ – GGAGCGAGATCCCTCCAAAAT – 3′ R: 5′ – GGCTGTTGTCATACTTCTCATGG – 3’

F: Forward primer; R: Reverse primer

### CCK-8 assay

OCI-Ly3 cells were cultured under standard conditions and transfected with different vectors until the cell confluence reached about 70%. The cells were then collected and seeded in the 96-well plates at a density of 1 × 10^4^ cells per well. A CCK-8 kit (Sigma) was used to assess cell proliferation according to the manufacturer’s protocol. Briefly, the CCK-8 solution (10 μl per well) was added to the wells and incubated with the cells for 2 h. The optical density (OD) values were determined at a wavelength of 450 nm and used to assess cell proliferation abilities.

### Flow cytometry analysis

DLBCL and healthy tissue samples were washed with phosphate-buffered saline (PBS), centrifuged at 800×g for 6 min, suspended in ice-cold 70% ethanol/PBS, centrifuged at 800×g for another 6 min, and suspended with PBS. An Annexin V-FITC/Propidium Iodide (PI) Apoptosis Detection Kit (Thermo Fisher Scientific) was used to determine the cell apoptosis ratio according to the manufacturer’s instructions. Briefly, the OCI-Ly3 cells were collected and suspended using 1× Annexin V Binding Buffer. After that, the Annexin V and PI staining solution was incubated with the cell suspensions at room temperature for 25 min without light. A BD LSR II Flow Cytometer (BD Biosciences) was used to determine the rate of apoptosis.

### Transwell invasion assay

A total of 2 × 10^5^ OCI-Ly3 cells/ml were plated in 200 μl of serum-free medium in the upper layer of a Corning Transwell chamber that was coated with Matrigel (BD Biosciences), while 800 μl medium supplemented with 10% FBS was added to the bottom chamber. After 24 h of incubation, the cells that had invaded were fixed with 4% paraformaldehyde (PA), stained with 0.1% crystal violet for 10 min and rinsed three times with PBS. For quantification, 5 randomly selected fields were analyzed.

### Elisa

The expressions of IL-10, IFN-γ and TNF-α were determined using enzyme-linked immune sorbent assay (ELISA). The cytokines secreted from T cells in a co-culture T cell–OCI-Ly3 cell system were analyzed in triplicate using the IL-10, IFN-γ and TNF-α ELISA kits (R&D Systems) according to the manufacturer’s instructions.

### Western blot

Total proteins were extracted from tissue samples and cells using RIPA lysis buffer (Beyotime Biotechnology) according to the manufacturer’s protocol. The BCA kit (Beyotime Biotechnology) was used to quantify protein concentrations. The target proteins were then separated via electrophoresis with 10% SDS-polyacrylamide gel (SDS-PAGE) and transferred to polyvinylidene fluoride (PVDF) membranes (Millipore). The membranes were incubated for 1 h at 37 °C with 5% skim milk diluted with TBS containing 0.1% Tween-20, then were incubated overnight at 4 °C with the primary rabbit antibodies against human PD-L1 (11,000, #13684, Cell Signaling Technology) and glyceraldehyde-3-phosphate dehydrogenase (GAPDH; 1:2500, ab9485, Abcam). Horseradish peroxidase-linked goat anti-rabbit IgG (12,000, ab205718, Abcam) was incubated with the membranes for 1 h at room temperature. An ECL Western blot detection kit (Bio-Rad) was employed to determine the optical density of the protein bands to evaluate the expression levels of the proteins.

### Dual-luciferase reporter gene assay

The PD-L1 fragment containing miR-214 binding sites was synthesized to generate wild-type (PD-L1-WT) or mutant-type PD-L1 (PD-L1-MUT). The PD-L1-WT and PD-L1-MUT fragments were subcloned into the Renilla luciferase gene Pgl3-Luciferase reporter vectors (Promega) to generate the pGL3-PD-L1 (WT) and pGL3-PD-L1 (MUT) vectors, respectively. After that, the vectors were co-transfected with miR-214 mimics or control mimics into HEK-293 T cells for 24 h. Finally, the cells were lysed using a Dual-Luciferase Assay Kit (Promega), and the luciferase activities were evaluated using the luminescence plate reader (Molecular Devices).

### Nude mouse model

The animal experiments were approved by the Ethical Committee of Liaocheng People’s Hospital. A total of 20 female BALB/c nude mice (4~5 weeks old) were randomly separated into two groups of 10 mice. The miR-214 mimic or control mimics was transfected into OCI-Ly3 cells and cultured in serum-free DMEM for 24 h. These OCI-Ly3 cells (1 × 10^7^) were subcutaneously inoculated into the mice when they were between 6 and 7 weeks old. After 4 weeks, all the mice were killed and the tumor tissues were collected for further experiments. Immunohistochemistry staining was used to observe the histomorphology and examine the expression of Ki-67 as described in an earlier study [22].

### Statistical analysis

All data are presented as the means ± standard deviation. The data were analyzed using SPSS 22.0 software (IBM). Spearman correlation analysis was performed to analyze the correlation between miR-214 and PD-L1 in DLBCL tissues using Graphpad Prism Version 8.0.2. *p* < 0.05 was considered statistically significant.

### Results

#### MiR-214 is downregulated in DLBCL tissues and cell lines

**Fig. 1:**
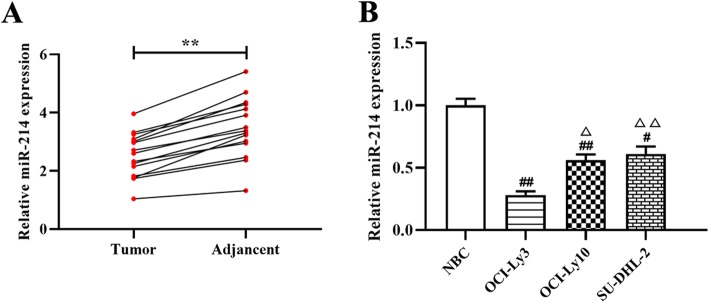
The expression of miR-214 in DLBCL tissues and cell lines. **a** and **b**—Quantitative RT-PCR was used to determine the expression levels of miR-214 in DLBCL tissues (**a**) and cell lines (**b**). **p < 0.01, compared with the adjacent normal tissues; ^#^*p* < 0.05, ^##^p < 0.01, compared with the normal B-cell line (NBC); ^△^*p* < 0.05, ^△△^*p* < 0.01, compared with the OCI-Ly3 cells

To explore the relationship between miR-214 and DLBCL development, quantitative RT-PCR was used to determine the expression level of miR-214 in DLBCL tissues (*n* = 15) and adjacent normal tissues (n = 15). As shown in Table 2, low miR-214 expression was positively associated with tumor size (*p* < 0.05), clinical stage (p < 0.05) and IPI scores (p < 0.05). The results also showed that the expression of miR-214 in DLBCL tissues was significantly lower than in the normal adjacent tissues (*p* < 0.01, Fig. 1a). Moreover, miR-214 was markedly downregulated in DLBCL cell lines compared with normal B-cell lines (NBC; p < 0.01, Fig. 1b), especially when comparing OCI-Ly3 cells (p < 0.01, Fig. 1b). Those results indicate that low expression of miR-214 may be related to the DLBCL progression. Based on these findings, OCI-Ly3 cells were chosen for subsequent experiments.

**Table 2:** The clinicopathological features of patients with DLBCL

Characteristic	Total number of patients	Expression of miR-214		*P* value
	15	High (*N* = 7)	^a^Low (*N* = 8)	
Age (years)				0.447
≥ 55	8 (53.33%)	3	5	
< 55	7 (46.67%)	4	3	
Gender				0.833
Male	9 (60.00%)	4	5	
Female	6 (40.00%)	3	3	
Tumor size (cm)				0.020
≥ 3	9 (60.00%)	2	7	
< 3	6 (40.00%)	5	1	
Clinical stage				0.036
I - II	5 (33.33%)	4	1	
III - IV	10 (66.67%)	2	7	
^b^LDH				0.782
High (≥ 300)	8 (53.33%)	4	4	
Low (< 300)	7 (46.67%)	3	4	
^c^IPI				0.013
Low (0–2)	4 (26.67%)	4	0	
High (≥ 3)	11 (73.33%)	3	8	

^a^The median of relative miR-214 expression level is 2.53, so the number of low miR-214 expression is 8 (< 2.53). ^b^LDH^:^ Lactate dehydrogenase; ^c^ IPI: International prognostic index

### Overexpression of miR-214 attenuates the malignant phenotype of OCI-Ly3 cells

**Fig. 2:**
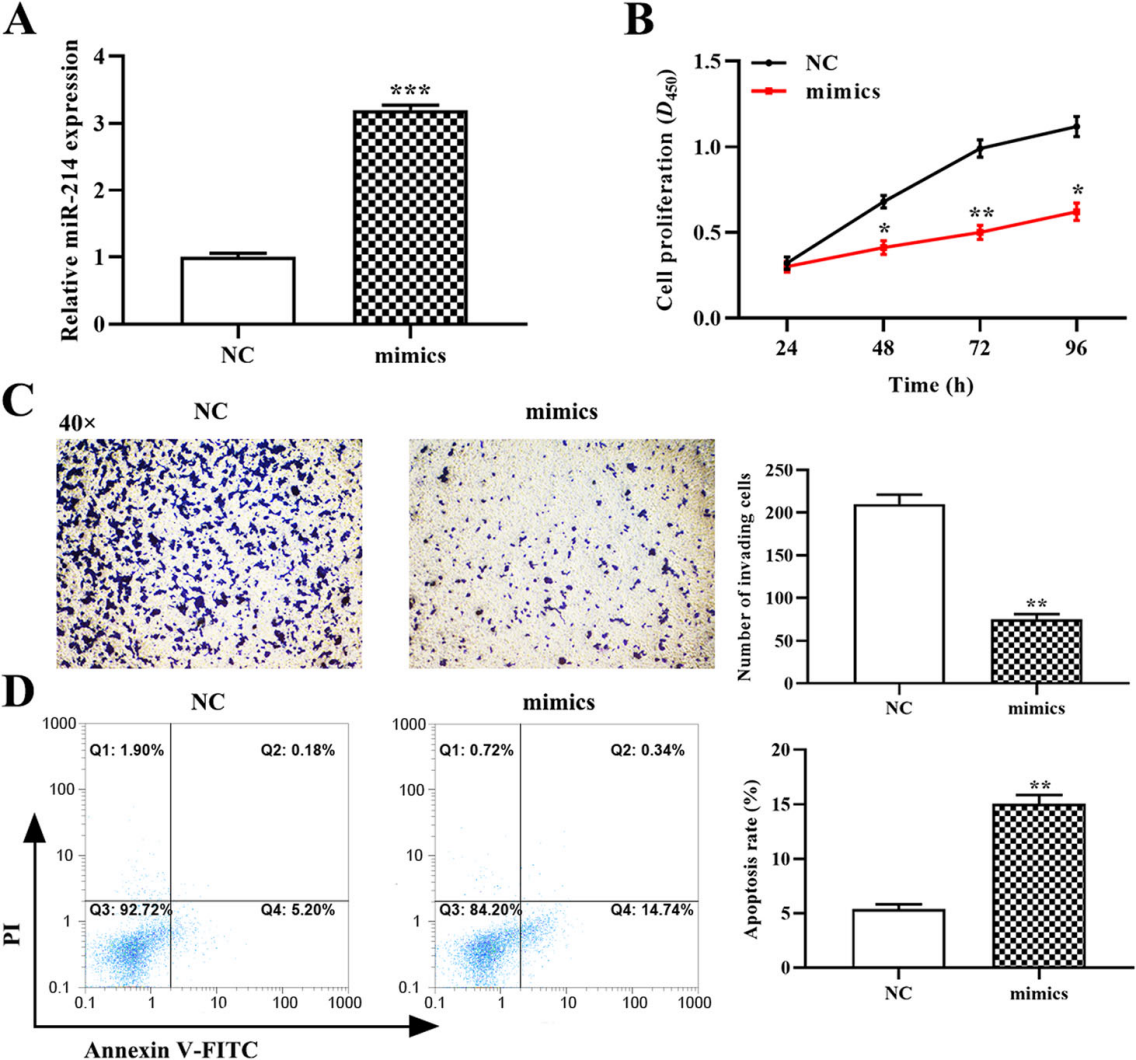
The impact of miR-214 on the proliferation, invasion and apoptosis of OCI-Ly3 cells. (**a**) The relative expression of miR-214 in cells transfected with an miR-214 mimic was determined using quantitative RT-PCR. (**b**) The proliferation of OCI-Ly3 cells was determined using the CCK-8 assay. (**c**) The invasion ability of OCI-Ly3 cells was assessed using a Transwell assay (magnification, × 40). (**d**) The rate of OCI-Ly3 cell apoptosis was measured using flow cytometry. *p < 0.05, **p < 0.01, ***p < 0.001, compared with the negative control (NC) group

Based on the downregulation of miR-214 in DLBCL tissues and cell lines, we attempted to explore the effect of miR-214 on OCI-Ly3 cell proliferation, invasion and apoptosis. OCI-Ly3 cells were transfected with the miR-214 mimic to assess the gain-of-function of miR-214. The expression of miR-214 was significantly enhanced in the miR-214 mimic group compared with the control group (*p* < 0.001, Fig. 2a), confirming successful transfection and enhancement of miR-214 expression.

Next, we investigated the impact of miR-214 upregulation on the proliferation and invasion of OCI-Ly3 cells using the CCK-8 and transwell assays. Overexpression of miR-214 significantly inhibited OCI-Ly3 cell viability compared with the negative control group (*p* < 0.05, Fig. 2b). Upregulated miR-214 also significantly suppressed the invasion capacity of OCI-Ly3 cells as compared to the negative control group (*p* < 0.01, Fig. 2c). Furthermore, Annexin V-FITC/PI double staining results showed that the increased expression of miR-214 contributed to inducing apoptosis of OCI-Ly3 cells (*p* < 0.01, Fig. 2d). These results strongly imply that overexpression of miR-214 suppresses cell proliferation and invasion and promotes apoptosis of OCI-Ly3 cells.

### MiR-214 negatively regulates the expression of PD-L1

**Fig. 3:**
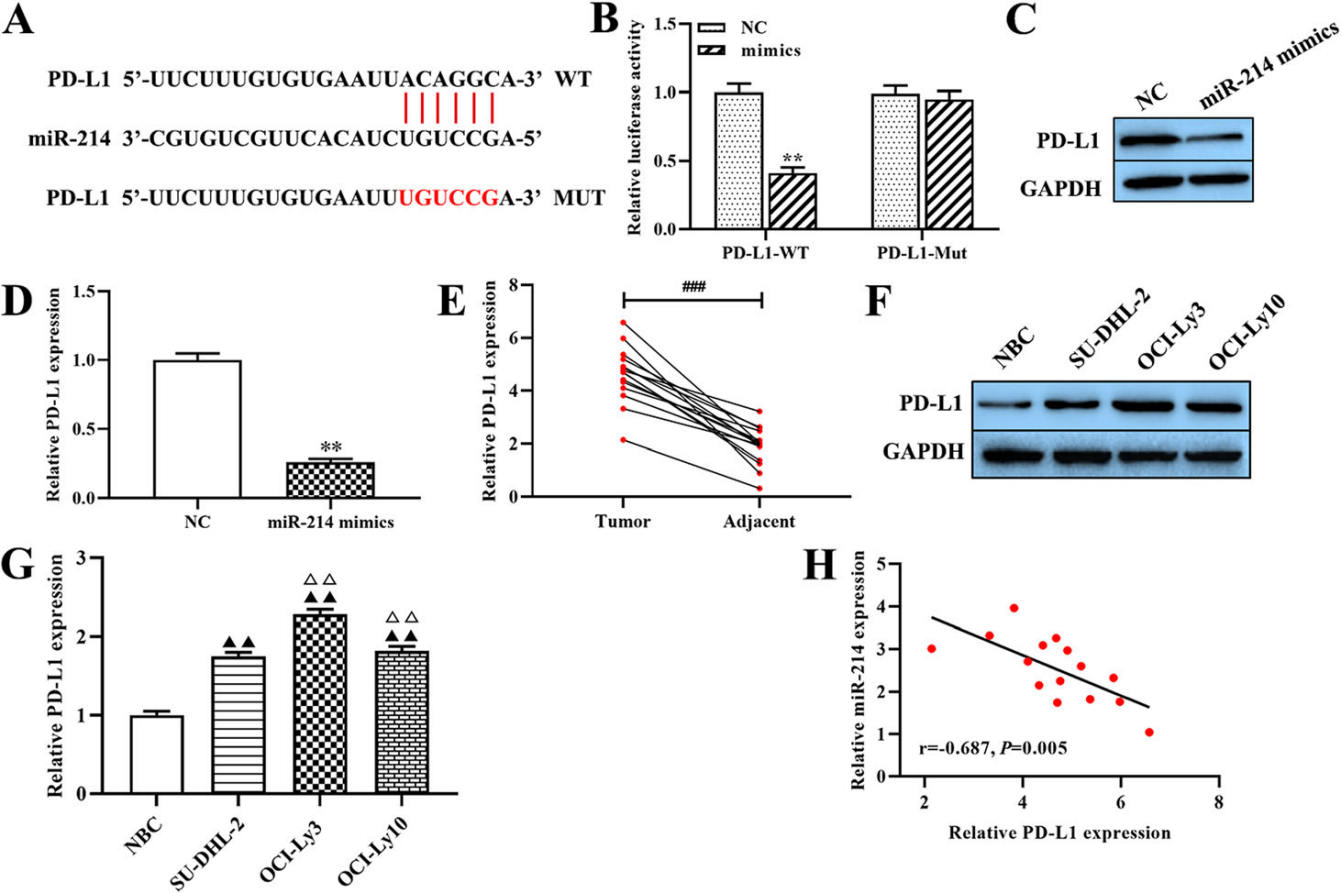
The regulatory relationship between miR-214 and PD-L1. (**a**) The bioinformatics analysis showed that miR-214 has a binding site with PD-L1. (**b**) The dual-luciferase reporter gene assay was used to verify the targeted relationship between miR-214 and PD-L1. (**c** and **d**) The expression of PD-L1 protein was determined using western blot. (**e**) Quantitative RT-PCR was used to determine the expression of PD-L1 in NSCLC tissues and adjacent tissues. (**f** and **g**) The expressions of PD-L1 in DLBCL cell lines were determined using quantitative RT-PCR. (**h**) The expression relationship between miR-214 and PD-L1 was evaluated using Spearman’s correlation analysis. **p < 0.01, compared with the NC group; ^###^p < 0.001, compared with the adjacent tissues; ^▲^p < 0.05, ^▲▲^p < 0.01, compared with the NBC group; ^△△^p < 0.01, compared with the OCI-Ly3 cells

The starBase database analysis revealed that miR-214 may target at PD-L1 directly (Fig. 3a). The dual-luciferase reporter gene assay result showed that co-transfection of miR-214 mimics and PD-L1-WT significantly decreased the luciferase activity (*p* < 0.01, Fig. 3b), but co-transfection of miR-214 mimics and PD-L1-MUT did not affect luciferase activity. Moreover, overexpression of miR-214 significantly decreased the expression levels of PD-L1 protein in OCI-Ly3 cells compared with the levels for the NC group (*p* < 0.01; Fig. 3c and d). Additionally, the expression of PD-L1 was markedly higher in DLBCL tissues than in the adjacent normal tissues (*p* < 0.001, Fig. 3e). The same as the result was obtained for PD-L1 protein expression in the DLBCL cell line compared to the normal B cell line (p < 0.01, Fig. 3f and g). Furthermore, Spearman’s correlation analysis revealed a marked negative correlation between the expressions of miR-214 and PD-L1 in DLBCL tissues (r = − 0.687, *p* < 0.01, Fig. 3h). These results show that PD-L1 is a target of miR-214 and that it has a lower expression in OCI-Ly3 cells.

### MiR-214 targets PD-L1 and attenuates the malignant phenotype of OCI-Ly3 cells

**Fig. 4:**
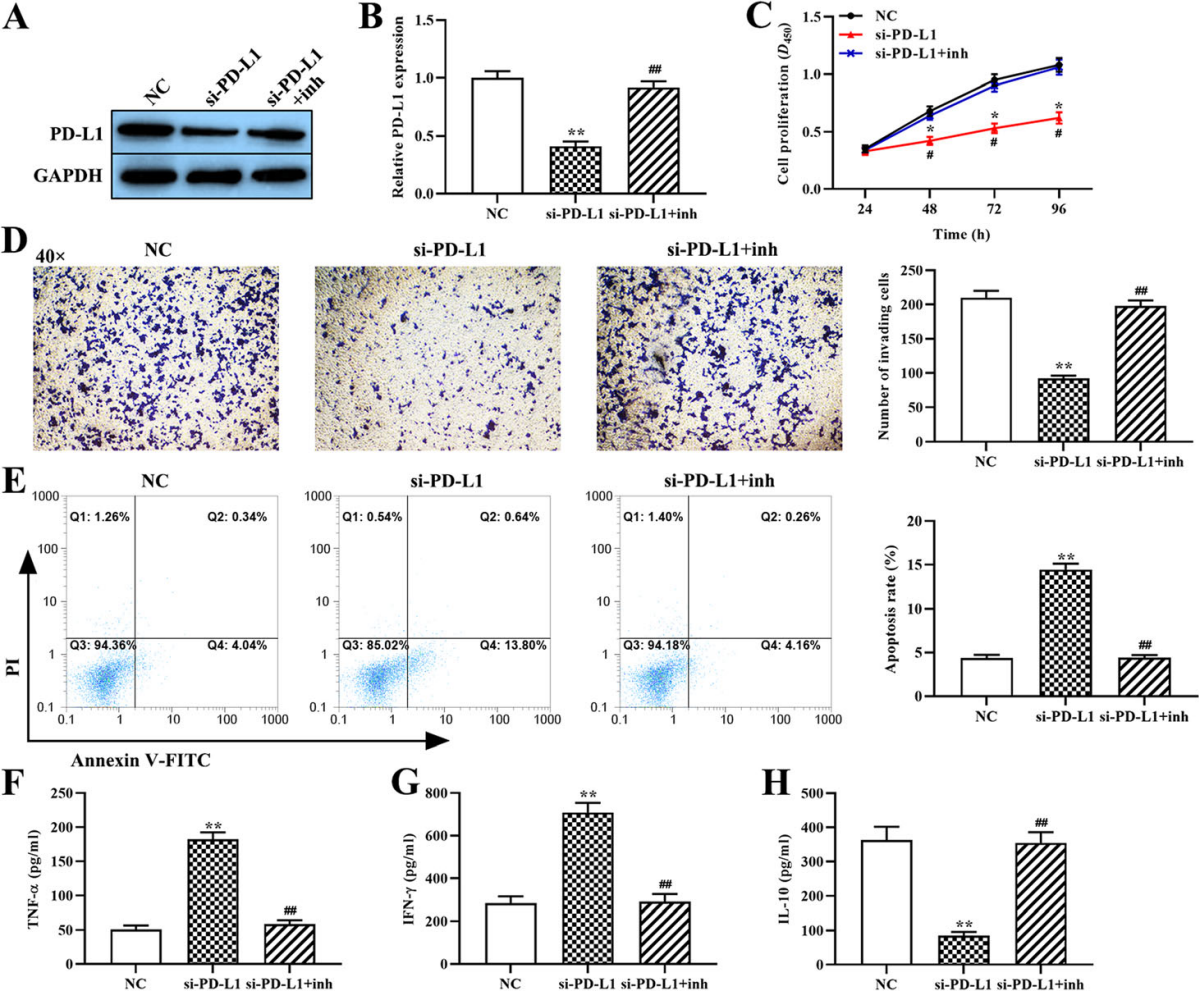
The effect of the miR-214–PD-L1 axis on the malignant behavior of OCI-Ly3 cells and on the cytokine secretion from T cells. (**a** and **b**) The expression of PD-L1 protein was determined using western blot. (**c**) The proliferation of OCI-Ly3 cells was determined using the CCK-8 assay. (**d**) The invasion ability of OCI-Ly3 cells was assessed using the Transwell assay (magnification, × 40). (**e**) The rate of OCI-Ly3 cell apoptosis was measured using flow cytometry. (**f** through **h**) The expression levels of TNF-α, IFN-γ and IL-10 were measured using ELISA. si-PD-L1: PD-L1 siRNA; si-PD-L1 + inh: PD-L1 siRNA + miR-214 inhibitor. *p < 0.05, **p < 0.01, compared with the NC group; ^#^p < 0.05, ^##^p < 0.01, compared with the si-PD-L1 group

We attempted to determine whether upregulation of miR-214 inhibits cell proliferation and invasion and induces apoptosis of OCI-Ly3 cells by targeting PD-L1. Crucially, western blot analysis results showed that PD-L1 knockdown decreased the levels of PD-L1 protein (*p* < 0.01, Fig. 4a, b), while co-transfection with miR-214 inhibitor restored them. Moreover, the CCK-8 and Transwell assays showed that knockdown of PD-L1 significantly decreased the proliferation (*p* < 0.05, Fig. 4c) and invasion (p < 0.01, Fig. 4d) of OCI-Ly3 cells compared with the negative control group. Furthermore, compared with the control group, PD-L1 knockdown increased the percentage of apoptotic OCI-Ly3 cells (*p* < 0.01, Fig. 4e). However, the effect of silencing PD-L1 on the behavior of OCI-Ly3 cells was reversed by co-transferction with the miR-214 inhibitor. These results suggest that miR-214 negative regulates PD-L1 to inhibit the proliferation and invasion and induce the apoptosis of OCI-Ly3 cells in vitro.

### MiR-214 targets PD-L1 to modulate cytokine secretion from T cells

To further determine the impact of the miR-214–PD-L1 axis on inflammatory cytokine secretion, we set up a co-culture system of OCI-Ly3 cells and T cells. The ELISA results show that knockdown of PD-L1 significantly increased the levels of IFN-γ and TNF-α compared with the control group (*p* < 0.01, Fig. 4f and g), but decreased those of IL-10 (*p* < 0.01, Fig. 4h). However, there was no significant difference between the cells co-transfected with miR-214 inhibitor and PD-L1 siRNA and those in the negative control group. This indicates that miR-214 targets PD-L1 to regulate the function of T cells and to further mediate the immune response of tumor cells.

### Upregulation of miR-214 suppresses DLBCL development in vivo

**Fig. 5:**
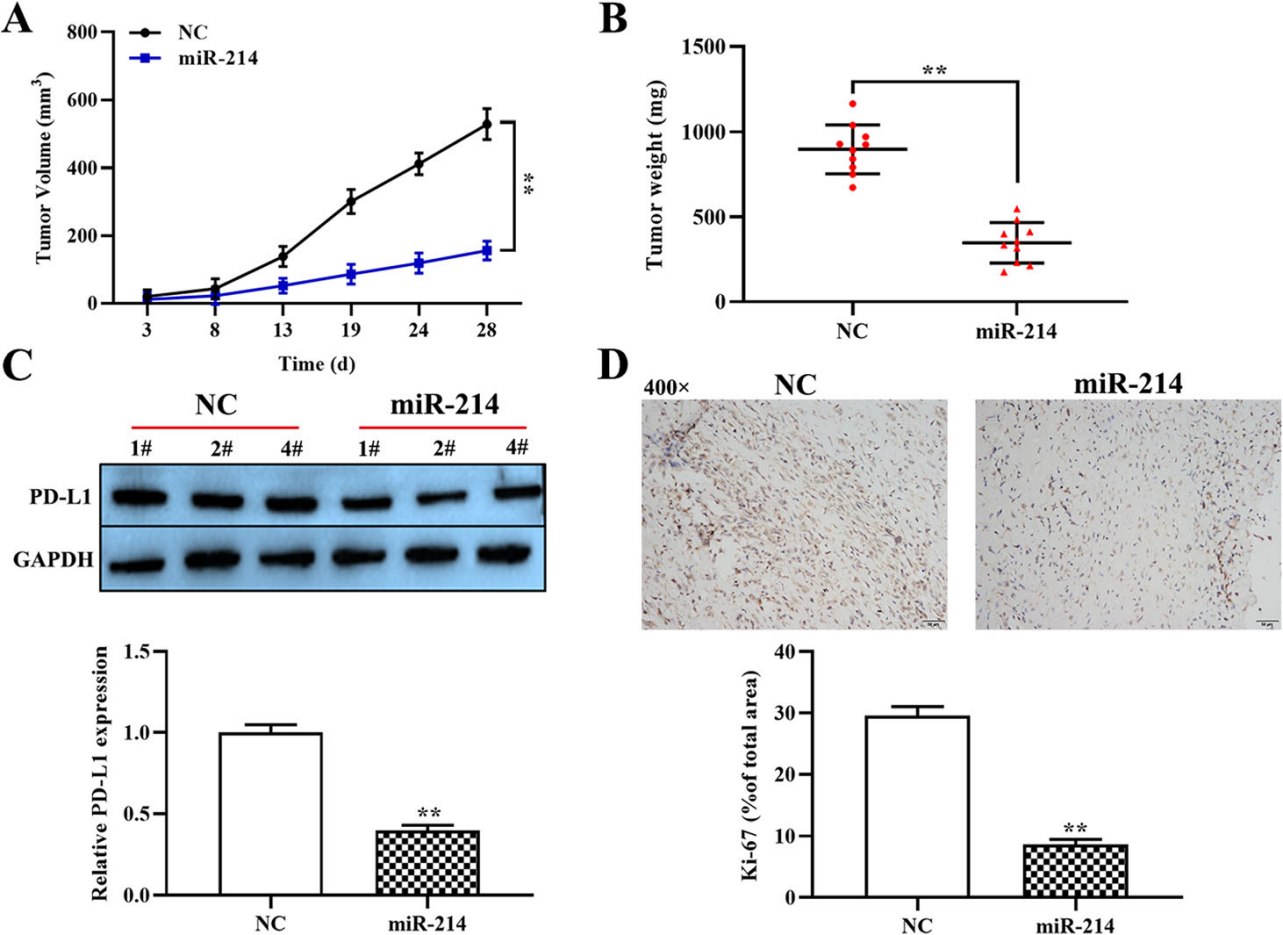
Effect of miR-214 overexpression on the progression of DLBCL in vivo. (**a**) The tumor volume curve of nude mice treated with miR-214 or NC was analyzed. (**b**) The tumor weight was measured in the transfected miR-214 or NC group. (**c**) The expression of PD-L1 protein in the tumor tissues was measured using western blot. (**d**) The expression of Ki-67 was determined in tumor tissues using immunohistochemistry (magnification, × 400). *p < 0.05, **p < 0.01, compared with the NC group

Having determined the impact of miR-214 on the proliferation, invasion and apoptosis of OCI-Ly3 cells, we attempted to examine the effect of miR-214 on the tumor growth of DLBCL in vivo. In a DLBCL mouse model with overexpression of miR-214, the tumor volume and weight were noticeably lower than in the negative control group (*p* < 0.01, Fig. 5a and b). Moreover, as in the cell lines, upregulation of miR-214 markedly decreased the expression of PD-L1 protein (p < 0.01, Fig. 5c). Immunohistochemistry results demonstrate that elevated miR-214 decreased the expression of Ki-67 in xenograft tumor tissues (p < 0.01, Fig. 5d). Our findings suggest that overexpression of miR-214 may restrict DLBCL progression by targeting PD-L1 in vivo.

## Discussion

This study focused on the molecular biomarkers in the development and progression of DLBCL. We found that miR-214 is significantly downregulated in DLBCL tissues and cell lines. We also established that it inhibits the proliferation and invasion and promotes the apoptosis of OCI-Ly3 cells by targeting PD-L1. Furthermore, in a co-culture system of OCI-Ly3 cells and T cells, knockdown of PD-L1 was found to increase the levels of IFN-γ and TNF-α, but to decrease the level of IL-10. This effect was reversed by knockdown of miR-214. Therefore, miR-214 plays an important role in regulating DLBCL progression and may be used as a target in DLBCL treatment.

Recent studies have established that miR-214 is downregulated in tumor tissues and cell lines, and that progression-free survival is shortened in patients with low expression of miR-214 [23]. MiR-214 participates in many cellular functions, including cell cycle control, DNA damage and repair, and gene transcription; but its aberrant expression affects cell migration, invasion and apoptosis of human malignant tumor cells by targeting downstream genes [24]. For example, overexpression of miR-214 inhibits proliferation and migration in hepatocellular carcinoma by targeting FOXM1 [25]. A low expression level of miR-214 is associated with lymph node metastasis, TNM stage and tumor size [26, 27]. Meanwhile, downregulation of miR-214 is associated with poor survival in leukemia [28, 29].

The miR-214 is not only significant for malignant tumor progression, but also plays an important role in regulating resistance to chemotherapy and radiotherapy in several tumors. For example, overexpression of miR-214 enhances the sensitivity of radiotherapy in colorectal cancer by decreasing ATG12-induced autophagy [30]. Elevated miR-214 reverses doxorubicin resistance in breast cancer by promoting cell apoptosis [31]. Our results demonstrate that overexpression of miR-214 significantly restricts the malignant behavior of OCI-Ly3 cells and decreases tumor growth in a xenograft mouse model.

PD-L1 monoclonal antibodies have recently been approved by the FDA for the United States, and they have been used in a variety of cancer therapies with good results [32, 33]. Importantly, PD-L1 acts as an immune checkpoint in cancer immunotherapy [34, 35]. Song et al. confirmed that the PD-1/PD-L1 pathway is an immune evasion mechanism associated with the progression of DLBCL [14]. Abnormal PD-L1 expression is used as a biomarker for early diagnosis and progression of several malignant tumors [36], such as lung cancer [37], thyroid cancer [38] and head and neck squamous cell carcinoma [39].

In addition, some studies found that miRNAs targets PD-L1 to regulate the proliferation, invasion and apoptosis of tumor cells and modulate the immune response. For example, miR-148a-3p overexpression inhibits the progression of colorectal cancer by targeting PD-L1 [40]. Repression of miR-940 promotes the proliferation and migration of gastric cancer by upregulating PD-L1 [41]. Overexpression of Epstein-Barr virus-encoded EBNA2 contributes to an increase in the immune escape of B-cell lymphomas through downregulation of the inhibitor effect of miR-34a on PD-L1 expression [42]. Here, we found that compared with the control group in a co-culture system of OCI-Ly3 cells and T cells, knockdown of PD-L1 significantly decreases the malignant behavior of OCI-Ly3 cells, increases the levels of IFN-γ and TNF-α, and decreases the level of IL-10.

In this study, we determined that overexpression of miR-214 could suppress the progression of DLBCL by targeting PD-L1 in vitro and in vivo. In addition, miR-214 targets PD-L1 to regulate the immune response of DLBCL by modulating the expressions of IL-10, IFN-γ and TNF-α. We hope these results will point the way to new molecular targets for the treatment of DLBCL and new biomarkers for its diagnosis and prognosis.

## Data Availability

The datasets and material used and/or analyzed during this study are available from the corresponding author on reasonable request.

## Abbreviations

CCK-8: Cell Counting Kit-8
DLBCL: diffuse large B-cell lymphoma
ELISA: enzyme-linked immune sorbent assay
miR-214: microRNA-214
MUT: mutant type
PD-L1: programmed death ligand-1
UTR: untranslated region
WT: wild type
